# Loss of visual feedback revealing motor impairment – an early symptom of Parkinson’s disease in two Irish farmers

**DOI:** 10.1186/s40734-016-0040-0

**Published:** 2016-08-01

**Authors:** Diana A. Olszewska, Conor Fearon, Tim Lynch

**Affiliations:** Dublin Neurological Institute at the Mater Misericordiae University Hospital, 57 Eccles Street, Dublin, Ireland

**Keywords:** Parkinson’s disease, Proprioception, Visual feedback

## Abstract

**Background:**

In the absence of visual feedback, humans depend upon proprioceptive information for reaching movements and coordination. Use of sensory information in order to assist movement is impaired in patients with early Parkinson’s disease (PD). It has been postulated that patients with PD compensate for this kinaesthetic deficit by relying on visual information.

**Case presentation:**

We report two farmers who first noticed symptoms of PD when working on the farm in situations requiring processing of the proprioceptive/kinaesthetic information in order to execute motor output in the absence of visual cues. A 68-year-old right-handed farmer had a 5-year history of left hand awkwardness. He first noticed the problem while performing artificial insemination in cattle using the recto-vaginal technique. He was diagnosed with PD 15 months after his initial symptoms and responded well to a combination of carbidopa-levodopa and ropinirole but has not returned to performing artificial insemination since. Clinical examination revealed asymmetric parkinsonism with normal sensation on gross neurological examination (including proprioception). A 60-year-old right-handed farmer had a 6-year history of difficulty manoeuvring his right hand whilst turning the calf during calving only when he did not have direct sight of his hand. 18 months later he developed right hand tremor and bradykinesia. He was diagnosed with PD 2 years following these initial symptoms. He had a good response to a combination of carbidopa-levodopa, rasagiline and ropinirole. He switched to using his left hand during calf delivery but is not as dextrous as previously. Clinical examination revealed parkinsonism, more marked in the right hand and normal sensation in all modalities.

**Conclusions:**

Although task-specific motor impairment could explain the symptoms of these patients, it is noteworthy that loss of visual feedback was central to both of these presentations. Given that early kinaesthetic deficits are known to be present in patients with PD, we postulate that removing visual feedback can expose such deficits in early PD, even when not detectable on routine examination. These two patients suggest that sensorimotor control can be impaired early in PD and may be the first symptom. Once the visual compensatory mechanism is not available, it becomes difficult or impossible to perform complex hand movements.

## Background

People with Parkinson’s disease (PwP) have motor and cognitive deficits but also difficulties in sensory processing [1, 2]. Pointing to remembered targets is impaired in PwP compared with controls, especially when visual feedback is not present (e.g. in dark conditions) [1]. In the absence of visual feedback, humans depend upon proprioceptive information for reaching, grasping movements and coordination [1, 2]. Use of sensory information (including proprioception) in order to assist movement is impaired in early PD [1, 2]. It has been proposed that PwP compensate for this proprioceptive/ kinaesthetic loss by relying on visual information.

We report two farmers who first noticed symptoms of PD when working on the farm in situations requiring processing of the proprioceptive information in order to execute motor output in the absence of visual cues.

## Case presentation

A 68-year-old right-handed farmer had a 5-year history of left hand awkwardness. He first noticed the problem while performing artificial insemination in cattle using the recto-vaginal technique [3]. The left hand is placed in the cattle’s rectum and used to manipulate the reproductive tract while the right hand holds the inseminating rod in the cervix [3]. He noticed difficulty manoeuvring his left hand when trying to locate, grasp, hold and push forward the cervix in order to insert the insemination rod. His left hand deteriorated over 3 months and he was unable to continue working. At the same time he had difficulty with holding a bucket. He did not have any difficulties with buttoning up, shaving, cutting food or any other activities at a time. One month later he developed a left hand tremor. He was diagnosed with PD 15 months after his initial symptoms and responded well to a combination of carbidopa-levodopa and ropinirole but has not returned to performing artificial insemination since. His vitamin B12 and folate were normal. Montreal Cognitive Assessment Test (MOCA) was 27/30.

His paternal first cousin and maternal second cousin had PD. Clinical examination revealed asymmetric parkinsonism with normal sensation (including proprioception).

A 60-year-old right-handed farmer had a 6-year history of difficulty manoeuvring his right hand whilst turning the calf during calving only when he did not have direct sight of his hand. The hand felt slow, clumsy and “like a vice”. He was unable to open his hand to grab the calf’s leg and turn the calf (same difficulty in lamb delivering). He was delivering approximately 10 calves per year for 30 years. At the time there was no difficulty with any other tasks such as: buttoning, shaving, cutting food.18 months later he developed right hand tremor and bradykinesia. Then he reported to have slight buttoning problems on the opposite side (when using the right hand). He was diagnosed with PD 2 years following these initial symptoms. He had a good response to a combination of carbidopa-levodopa, rasagiline and ropinirole. He switched to using his left hand during calf delivery but is not as dextrous as previously. Clinical examination revealed parkinsonism, more marked in the right hand and normal proprioception. His B12 and folate were normal.

## Conclusion

Our first patient also reported difficulty holding a bucket at the same time as artificial insemination difficulty; however his dexterity problem was greatest during insemination. It is possible that loss of strength was the reason. However strength was normal on examination. It is also possible that bradykinesia related-loss of motor dexterity was the cause of difficulties. However, they managed other manual tasks well particularly with visual input. Moreover the initial symptom was specific to a task (artificial insemination or calf/lamb delivery) where they relied on kinesthesia alone without visual input. These are less likely to be due to bradykinesia as both patients were not bradykinetic at a time when performing any other simple or complex tasks. Although task-specific motor impairment could explain the symptoms of these patients, it is noteworthy that loss of visual feedback was central to both of these presentations. Given that early proprioceptive/kinaesthetic deficits are known to be present in PwP, we postulate that removing visual feedback can expose such deficits in early PD, even when not detectable on routine examination. These subtle kinaesthetic deficits may not be evident on clinical examination, but have been exposed in an experimental setting using pointing to targets with and without visual feedback [1]. Kinaesthetic deficits occur in the early PD stages and are compensated by reliance on visual cues [1]. This may be why visual cues improve gait and freezing in PwP [4]. These kinaesthetic deficits are often not detectable on routine physical examination. PwP point to targets less accurately than healthy controls, especially when visual feedback is absent. Larger errors in the dark suggest that PwP are less able to use proprioceptive information when the visual cue is lost [1]. As the disease progresses the benefit of this visual feedback is lost [1].

The basal ganglia receive visual and kinaesthetic inputs and may have a role in multisensory integration. Dopamine depletion disrupts this integration and patients become dependent on external stimuli to initiate motor output. Animal studies have shown that kinesthetic processing deficits correlate with degree of dopamine loss in basal ganglia [2].

With minor dopamine loss, these impairments can be compensated for by integrating visual information [2]. Movements execution becomes more complex when proprioceptive information is lost and patients are forced to depend on visual cues [2]. Basal ganglia receive minor afferents from visual cortex, but the crucial area for visuo-motor control is located in cerebellar and parietal circuits which are spared in PD providing a compensatory mechanism for visual control of movement [2].

Both of our patients improved on dopamine replacement therapy from the motor point of view; however their ability to perform these specific tasks did not improve (one has never returned to work, the other had to change the technique). This is consistent with the reports that dopamine replacement therapy is effective in improving many symptoms; however the effects on proprioception are not fully understood and levodopa therapy may even have an added detrimental effect on proprioception [2].

These two patients suggest that sensorimotor control can be impaired early in PD and may be the first symptom. Once the visual compensatory mechanism is not available, it becomes difficult or impossible to perform complex hand movements.

Possible explanations for the difficulties our patients experienced include subtle proprioceptive deficits not appreciable on routine physical examination or cortical sensory processing deficits. Either way these deficits were exposed in the setting of loss of visual feedback. Since compensation using visual cues can occur in early PD, motor difficulties in setting of loss of visual feedback may be an important early clue to diagnosis.

## Abbreviations

PD, Parkinson’s disease; PwP, People with Parkinson’s disease
